# RNaseH1 regulates TERRA-telomeric DNA hybrids and telomere maintenance in ALT tumour cells

**DOI:** 10.1038/ncomms6220

**Published:** 2014-10-21

**Authors:** Rajika Arora, Yongwoo Lee, Harry Wischnewski, Catherine M. Brun, Tobias Schwarz, Claus M. Azzalin

**Affiliations:** 1 Institute of Biochemistry, Eidgenössische Technische Hochschule Zürich (ETHZ), Zürich CH-8093, Switzerland

## Abstract

A fraction of cancer cells maintain telomeres through the telomerase-independent, ‘Alternative Lengthening of Telomeres’ (ALT) pathway. ALT relies on homologous recombination (HR) between telomeric sequences; yet, what makes ALT telomeres recombinogenic remains unclear. Here we show that the RNA endonuclease RNaseH1 regulates the levels of RNA–DNA hybrids between telomeric DNA and the long noncoding RNA TERRA, and is a key mediator of telomere maintenance in ALT cells. RNaseH1 associated to telomeres specifically in ALT cells and its depletion led to telomeric hybrid accumulation, exposure of single-stranded telomeric DNA, activation of replication protein A at telomeres and abrupt telomere excision. Conversely, overexpression of RNaseH1 weakened the recombinogenic nature of ALT telomeres and led to telomere shortening. Altering cellular RNaseH1 levels did not perturb telomere homoeostasis in telomerase-positive cells. RNaseH1 maintains regulated levels of telomeric RNA–DNA hybrids at ALT telomeres to trigger HR without compromising telomere integrity too severely.

## Abstract (web summary)

A subset of cancers maintains telomere length independently of telomerase by activating alternative lengthening of telomeres (ALT) pathways. Here the authors show that RNaseH1 modulates telomeric homologous recombination frequencies in ALT cells by regulating the levels of RNA–DNA hybrids between TERRA and telomeric DNA.

## Graphical abstract

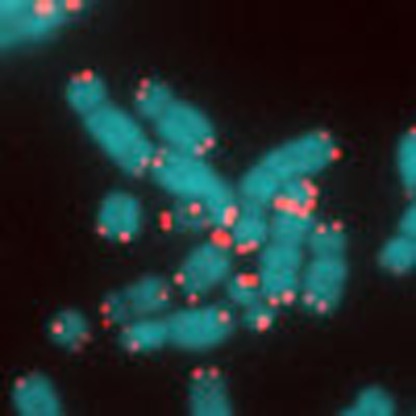

Telomeres, the heterochromatic nucleoprotein complexes located at the ends of linear eukaryotic chromosomes, allow cells to distinguish between natural chromosome ends and accidental DNA double-stranded breaks, thereby avoiding unwanted DNA repair and degradation^1^,^2^. Telomeres also set the lifespan of human somatic cells by triggering an irreversible cell-cycle arrest when they become ‘critically short’ upon successive population doublings, in a process known as cellular senescence^3^. The core telomeric structure comprises arrays of tandem DNA repeats (5′-TTAGGG-3′ in vertebrates), a telomere-specific multiprotein complex dubbed ‘shelterin’, and the long noncoding RNA (lncRNA) telomeric repeat-containing RNA (TERRA)^1^,^2^,^4^,^5^. DNA-dependent RNA polymerase II (RNAPII) uses the C-rich telomeric strand as a template to produce G-rich TERRA molecules, which remain associated with telomeric heterochromatin post transcriptionally^6^,^7^. In humans, TERRA is transcribed from CpG dinucleotide-containing promoters located at least on half of human subtelomeres. TERRA promoter CpG dinucleotides are methylated by the DNA methyltransferases DNMT1 and DNMT3b, and simultaneous gene deletion of the two enzymes leads to de-repression of TERRA transcription^8^.

Because cancer cells rely on immortality to propagate indefinitely, they must acquire at least one telomere lengthening mechanism to counteract replication-dependent telomere shortening and senescence. While the majority of cancer cells reactivate the specialized reverse transcriptase telomerase, 10–15% of cancers utilize the so-called ‘Alternative Lengthening of Telomeres’ (ALT) pathway to counteract telomere loss^9^,^10^. ALT has been documented in various aggressive cancers including sarcomas, gastric carcinomas, central nervous system malignancies and bladder carcinomas, as well as in a subset of *in vitro* immortalized cells lines^9^,^10^. ALT telomeres possess a number of peculiar traits commonly used as ALT-associated markers: (i) telomeres of very heterogeneous length at different chromosome ends; (ii) association of multiple telomeres in nuclear bodies containing promyelocytic leukaemia (PML) forming the so-called ALT-associated PML bodies (APBs); (iii) abundant extra-chromosomal telomeric DNA in the form of double-stranded telomeric circles (t-circles), partially single-stranded circles (C- and G-circles) and linear double-stranded DNA; (iv) elevated rates of telomeric sister chromatid exchanges^9^,^10^. Recently, accumulating evidence also indicates that ALT cells are characterized by elevated levels of TERRA (refs 6, 7, 11, 12).

Although the molecular details of ALT remain to be fully elucidated, it is commonly accepted that ALT telomeres are maintained by mechanisms relying on homologous recombination (HR) between telomeric repeats. Consistently, several HR proteins have been found to localize to ALT telomeres and their functional inactivation leads to loss of telomeric sequences and eventually cell growth arrest and death^9^,^10^. It has been suggested that telomeric sister chromatid exchanges could sustain elongation of one sister telomere at the expense of shortening of the other one or that telomere elongation is accomplished through break-induced replication, a HR-based repair mechanism that uses a homologous donor template to synthesize up to several kilobases of new DNA starting from a break site. It is also possible that HR could engage between telomeres and extra-chromosomal telomeric DNA (refs 9, 10). Still, it is completely unknown what molecular features render ALT telomeres recombinogenic. We show here that TERRA plays a crucial role in this process by forming RNA–DNA hybrids with the telomeric C-rich DNA strand. TERRA hybrids are finely regulated by the RNA endonuclease RNaseH1, which associates to telomeres in ALT but not in telomerase-positive cells. Altering the cellular levels of RNaseH1 impacts on hybrid formation as well as on the recombinogenic potential of ALT telomeres. By addressing a longstanding question in telomere biology, we anticipate that our studies will pave the way for the development of anti ALT treatments to be used alone or in combination with anti-telomerase drugs.

## Results

### TERRA and telomere transcription are elevated in ALT cells

**Figure 1:**
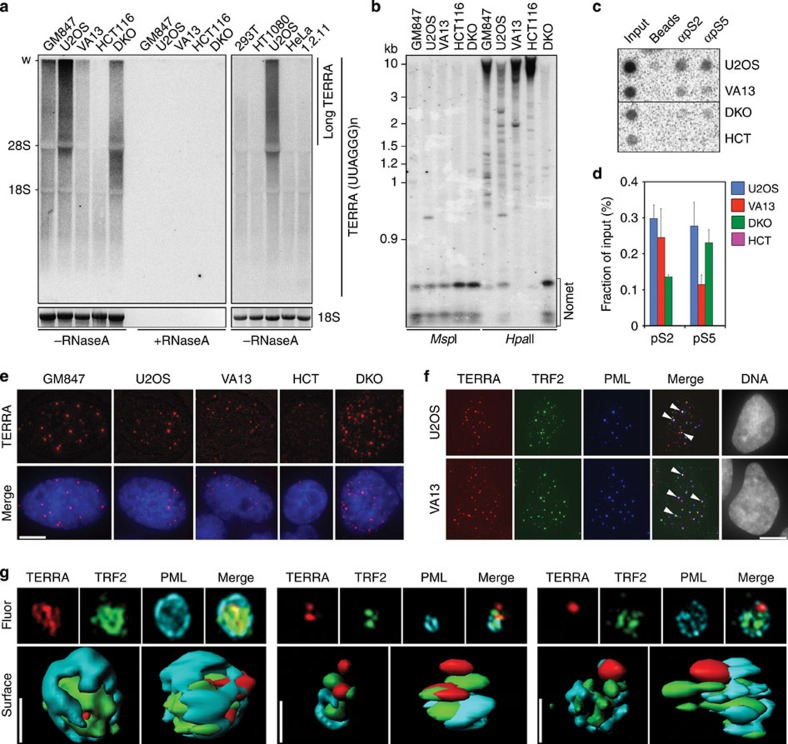
TERRA and telomere transcription in ALT cells. (**a**) TERRA northern blot hybridizations of RNA from the indicated cell lines (VA13: WI-38 VA13; 1.2.11: HeLa 1.2.11) pre-treated with RNaseA or left untreated. Ethidium bromide stained 18S ribosomal RNA (rRNA) is shown to control for loading. Long TERRA molecules comprised between the wells of the gels (w) and 28S rRNA are indicated. (**b**) TERRA CpG-island promoter methylation analysis of the indicated cell lines. Genomic DNA was digested with the methylation sensitive restriction enzyme *Msp*I or its methylation insensitive isoschizomer *Hpa*II. DNA was hybridized using a radioactively labelled probe detecting TERRA promoter CpG-island repeats. Nomet: fragments corresponding to unmethylated restriction products. (**c**) Dot blot hybridization of DNA immunoprecipitated with antibodies against phosphorylated Serines S2 and S5 of RNA polymerase II C-terminal domain. Hybridizations were performed with a telomeric probe. Quantifications are shown at the bottom. (**d**) Bars and error bars are averages and s.d. from three independent experiments. (**e**) Examples of TERRA FISH in the indicated cells. TERRA is shown in red, DAPI-stained DNA in blue. Scale bar, 9 μm. (**f**) IF/FISH experiments in the indicated cell lines. TERRA is in red, TRF2 in green and PML in blue. In the merge panels, arrowheads point to nuclear foci where the three factors co-localize. Scale bar, 9 μm. (**g**) Information surface at 0.01 μm detail level of three TERRA-containing APBs. TERRA is in red, TRF2 in green and PML in cyan. Images were generated with Three-Dimensional Structured Illumination Microscopy (3D-SIM). Scale bars, 0.4 μm.

To analyze TERRA cellular levels in different cell lines we performed northern blot analysis of total RNA following a protocol that assures efficient transfer of high molecular weight RNA species^13^. Consistently with previous reports^6^,^7^,^11^,^12^, we found that TERRA was more abundant in the three ALT cell lines GM847, U2OS and WI-38 VA13 than in telomerase-positive HeLa, HT1080, HEK 293T and HCT116 cells (Fig. 1a). Moreover, we detected extremely long TERRA transcripts comprised between the 28S ribosomal RNA and the wells of the gel specifically in ALT cells (Fig. 1a). In U2OS cells ectopically expressing telomerase (U2OS-T), high molecular weight TERRA signal was further increased as compared with parental U2OS cells (Supplementary Fig. 1a). Thus, the appearance of long TERRA species in ALT cells does not derive from a lack in telomerase activity. Telomerase-positive HeLa1.2.11 cells, which have very long telomeres, did not accumulate long TERRA molecules (Fig. 1a), indicating that the sole presence of long telomeres in ALT cells is not sufficient to explain the existence of long TERRA species. Elevated TERRA levels and long TERRA molecules were also present in telomerase-positive, HCT116-derived cells double knocked out (DKO) for DNA methyltransferase 1 and 3b genes (DKO cells; Fig. 1a). DKO cells carry de-methylated and de-repressed TERRA CpG-island promoters^8^, suggesting that the increase in TERRA levels observed in ALT cells could derive, at least in part, from increased RNAPII-mediated transcription. Indeed, TERRA CpG-island promoters were hypomethylated and RNAPII bound more avidly to telomeric repeats in ALT cells (Fig. 1b–d). These observations are in line with the notion that chromatin is altered at ALT telomeres^12^,^14^.

We also performed RNA fluorescence *in situ* hybridization (FISH) experiments combined with indirect immunofluorescence (IF) to simultaneously detect TERRA, PML and the shelterin component TRF2. Images were acquired using canonical or super-resolution microscopy. In U2OS cells, 8% of TERRA foci colocalized with APBs (defined as foci containing both PML and TRF2), while 60% of APBs contained TERRA. In U2OS-T cells, 4% of TERRA foci colocalized with APBs and the fraction of APBs containing TERRA lowered to 12%, suggesting that telomerase restricts TERRA localization to APBs. Finally, in WI-38 VA13 cells, 37% of TERRA foci were in APBs and 42% of APBs contained detectable TERRA (Supplementary Fig. 1b–d). Altogether these data indicate that TERRA is a novel component of APBs.

### TERRA transcription induces telomere instability in ALT cells

**Figure 2:**
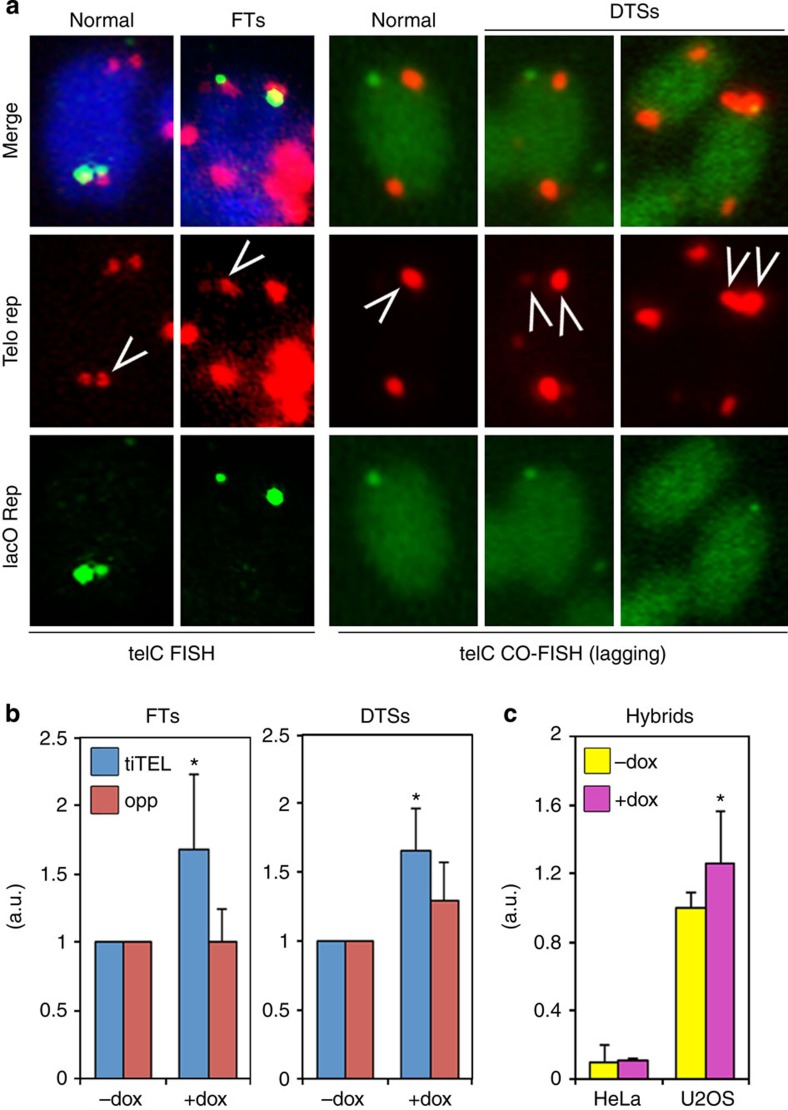
Transcription induction promotes telomeric aberrations and RNA–DNA hybrids in ALT cells. TiTEL transcription was induced for 48 h before performing FISH and CO-FISH experiments. (**a**) Examples of normal and aberrant tiTELs (arrows) detected by FISH. TiTEL chromosome ends were visualized with lacO PNA probes (green), telomeric repeats with telomeric C-rich probes (red), DNA was counterstained with DAPI (blue). (**b**) Quantification of aberrations at tiTELs and telomeres at opposite chromosome ends (opp). Untreated samples were set to 1. Bars and error bars are averages and s.d. from at least three experiments where a total of at least 180 tiTEls and opposite chromosome ends were scored. *P*-values were computed using the Student’s *t*-test. *P<0.05 (+dox versus −dox). (**c**) Quantification of telomeric hybrids at tiTELs in U2OS and HeLa cells measured by DIP using the S9.6 antibody. U2OS untreated samples were set to 1. Bars and error bars are averages and s.d. from at least three experiments. *P*-values were computed using the Student’s *t*-test. *P<0.05 (+dox versus −dox).

To probe the functional relevance of telomere transcription in ALT, we generated a U2OS-derived cell line carrying a transcriptionally inducible telomere (tiTEL) whose transcription can be stimulated experimentally from a doxycycline (dox) responsive cytomegalovirus promoter (Supplementary Fig. 2). Dox treatment for 48 h led to an approximate twofold increase in transcriptionally inducible TERRA (tiTERRA) levels and a concomitant 1.5–2-fold increase in fragile tiTELs, improperly replicated telomeres visualized as shredded or multiple signals in metaphase FISH experiments^15^ (Fig. 2a,b and Supplementary Fig. 2c). Dox did not alter telomere fragility at chromosome ends opposite to tiTELs (Fig. 2b) nor did it affect tiTEL stability in a previously established HeLa-derived cell line^16^ despite a ~10-fold induction of tiTERRA (Supplementary Fig. 2c). Chromosome orientation FISH (CO-FISH) in induced U2OS tiTEL cells disclosed a 1.5–2-fold increase in double tiTEL signals, indicative of HR involving tiTEL sequences, as compared with uninduced cells (Fig. 2a,b). On the contrary, dox treatment did not significantly alter the incidence of double telomeric signals (DTSs) at chromosome ends opposite to tiTELs (Fig. 2b). Thus, TERRA transcription appears to promote telomere instability and recombination in an ALT background. Since TERRA levels are elevated in ALT cells, we tested whether ALT cells are characterized by increased telomere instability by performing PNA FISH on metaphase chromosomes. Indeed, we found that ALT cells displayed higher incidence of fragile telomeres (FTs) and telomere free ends (TFEs) than telomerase-positive cells (Supplementary Fig. 3). In U2OS-T cells, TFEs, but not FTs, were less frequent than in parental U2OS cells and the other ALT cell lines (Supplementary Fig. 3). Hence telomerase, while replenishing very short telomeres, is not able to fully revert ALT-associated telomere instability.

### TERRA forms RNA–DNA hybrid structures in human cancer cells

We previously proposed that aberrant TERRA localization to telomeres could harm telomere stability by impairing telomere replication through formation of RNA–DNA hybrids with the telomeric C-rich DNA strand, which templates both TERRA transcription and leading-strand replication^17^,^18^. TERRA:telomere hybrids (telomeric hybrids) have been recently reported in budding yeast and unresolved hybrids can induce genome instability by impairing DNA replication and promoting recombination^19^,^20^,^21^,^22^,^23^. We measured RNA–DNA hybrids at tiTELs by DNA immunoprecipitation (DIP) with the RNA–DNA hybrid specific, sequence-independent S9.6 antibody^24^ followed by real-time PCR. Hybrids involving tiTEL sequences were more abundant in U2OS cells than in HeLa cells already in uninduced conditions, suggesting that ALT telomeres are more prone to accumulating hybrids. Moreover, transcription induction led to a mild yet highly reproducible increase in hybrids at U2OS but not HeLa tiTELs (Fig. 2c). These findings prompted us to assess the existence of hybrids at natural telomeres in different cell lines. Telomeric RNA–DNA hybrids were detected in all tested telomerase-positive and ALT cell lines by DIP. Hybrids were more abundant at TERRA CpG-island promoter-containing chromosome ends (10q and 15q) than at XpYp subtelomeres, which are devoid of canonical promoters^8^ (Supplementary Fig. 4a,b). We next performed TERRA FISH on fixed cells previously treated with recombinant RNaseH or left untreated and found diminished TERRA signals in all tested cells upon treatment (Supplementary Fig. 4c,d). RNaseH sensitivity was particularly robust in U2OS and VA13 cells, where 70 and 60% of total TERRA signal disappeared upon treatment, respectively (Supplementary Fig. 4c,d). Thus, human cancer cells contain TERRA:telomeric DNA hybrids and these structures might be more abundant in some ALT cells.

To test the ability of telomeric DNA to generate hybrid structures, we *in vitro* transcribed plasmids containing ~800 bp long telomeric tracts using T7 polymerase. Strong accumulation of RNA–DNA hybrids occurred when transcription proceeded in the direction producing TERRA-like molecules, while hybrids were approximately 2- and 10-fold lower when templates were an inverted telomeric stretch or empty plasmid DNA, respectively (Supplementary Fig. 5a). Also, yields of TERRA-like RNA were much lower than antisense-TERRA or control Luciferase RNA (Supplementary Fig. 5b). We conclude that TERRA transcription represents a challenge for RNA polymerases and promotes RNA–DNA hybrid formation, suggesting that telomeric hybrids detected *in vivo* could arise at least in part cotranscriptionally. This notion is further supported by the fact that tiTERRA induction increases RNA–DNA hybrids at tiTELs in U2OS cells (Fig. 2c).

### RNase H1 restricts telomeric hybrids and C-rich ssDNA in ALT cells

**Figure 3:**
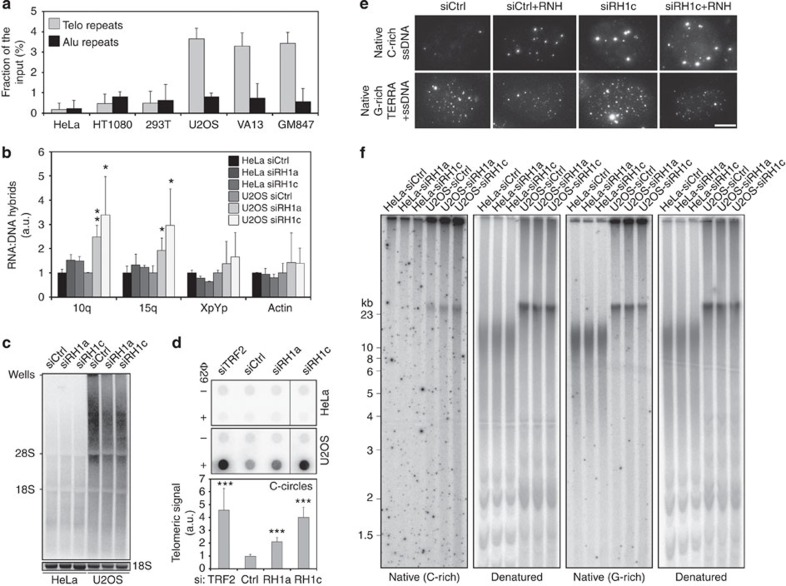
RNaseH1 functions at ALT telomeres. (**a**) Quantification of ChIP dot blot experiments performed in the indicated cell lines using antibodies against endogenous RNaseH1. DNA was first hybridized with telomeric probes and then with Alu repeat probes for specificity. Immunoprecipitated DNA is expressed as fraction of input DNA after subtraction of the background signal associated to control ChIPs using normal IgGs. Bars and error bars are averages and s.d. from three to five experiments. (**b**) Quantification of telomeric hybrids from the indicated chromosome ends and actin locus measured in S9.6 DIP experiments using cells transfected for 72 h with control siRNAs (siCtrl, set to 1) or siRNAs against RNaseH1 (siRH1a and c). Hybrids are expressed as fractions of the input material after subtraction of values from control immunoprecipitations with only beads. Bars and error bars are averages and s.d. from at least three experiments. *P*-values were computed using the Student’s *t*-test. *P<0.05, **P<0.005 (siRH1a or c versus siCtrl). (**c**) TERRA northern blot using total RNA from RNaseH1-depleted cells. (**d**) C-circle assays in siRNA transfected HeLa and U2OS cells. Anti-TRF2 siRNA was used as a positive control. Dot blots were hybridized with a C-rich telomeric probe in native conditions. Negative controls are reactions performed in the absence of the Ф29 enzyme. Quantification of C-circle signals for U2OS cells is at the bottom where siCtrl was set to 1. Bars and error bars are averages and s.d. from at least three experiments. *P*-values were computed using the Student’s *t*-test. ***P<0.0001 (siRH1a, siRH1c or siTRF2 versus siCtrl). (**e**) Native FISH experiments in U2OS cells transfected with the indicated siRNAs and treated or not with RNaseH (RNH) *in vitro* prior to hybridization. In the upper panels, C-rich ssDNA is detected with a G-rich telomeric probe; in the lower panels TERRA and telomeric G-rich ssDNA are detected simultaneously with a C-rich telomeric probe. Scale bar, 9 μm. (**f**) In-gel telomere restriction fragment analysis using digested genomic DNA prepared from cells depleted for RNaseH1. Hybridizations were first performed in native conditions using either a telomeric G-rich oligonucleotide probe (to detect single-stranded telomeric C-rich DNA; first panel from the left) or a telomeric C-rich oligonucleotide probe (to detect single-stranded telomeric G-rich DNA; third panel from the left). The same gels were denatured and re-hybridized with a double-stranded telomeric probe.

**Figure 4:**
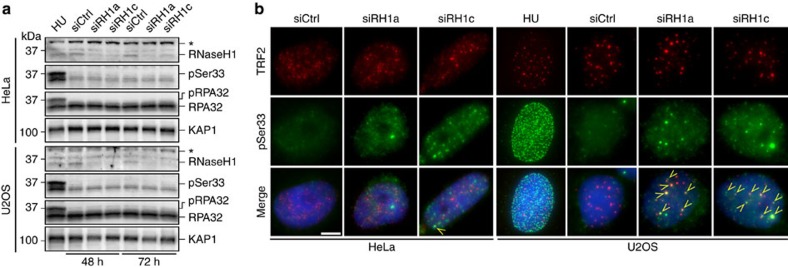
RNaseH1 depletion leads to RPA activation at ALT telomeres. (**a**) HeLa and U2OS cells were transfected with the indicated siRNAs and 48 and 72 h later protein extracts were prepared. Western blot analysis was performed using antibodies against RNaseH1, RPA32 phosphorylated at Serine 33 (pSer33), total RPA32 and KAP1 (loading control). The asterisk indicates a cross-reacting band. Cells treated for 6 h with 5 mM hydroxyurea (HU) were used as controls for pSer33 activation. (**b**) SiRNA transfected cells were subjected to indirect immunofluorescence using antibodies against TRF2 (to visualize telomeres; red) and pSer33 (green). DNA was counterstained with DAPI (blue). Arrows point to examples of pSer33 foci co-localizing with TRF2 (TIFs). Scale bar, 9 μm. Cells treated for 6 h with 5 mM HU were used as controls for pSer33 activation.

In search of cellular regulators of telomeric hybrids, we first transfected U2OS and HeLa cells with short interfering RNAs (siRNAs) against the shelterin components TRF1 and TRF2. Protein depletions were very efficient, generated telomeric DNA damage and consistent with published data^25^,^26^, TRF2 depletion increased TERRA levels at least in U2OS cells (Supplementary Fig. 6a,b). Yet, telomeric hybrid levels were not substantially altered upon TRF1 and TRF2 depletion in either cell line (Supplementary Fig. 6c). We then turned to the RNA–DNA hybrid-specific endonuclease RNaseH1 (ref. 27). We first tested its ability to localize to telomeres *in vivo* using chromatin immunoprecipitation (ChIP) and found that endogenous RNaseH1 was highly enriched at telomeric repeats over Alu repeats in U2OS, WI-38 VA13 and GM847 cells but not in telomerase-positive cells (Fig. 3a), suggesting an exclusive role for this enzyme at ALT telomeres. We then depleted RNaseH1 using two independent siRNAs (siRH1a and siRH1c), both leading to extensive depletion of the endogenous protein as compared with cells transfected with control siRNAs (siCtrl; Fig. 4a). RNaseH1 depletion stabilized telomeric hybrids in U2OS but not in HeLa cells (Fig. 3b). Hybrids mostly accumulated at TERRA promoter-containing chromosome ends, while no statistically significant effect was observed on hybrids at XpYp subtelomeres (Fig. 3b). As a non-telomeric control, we also measured hybrids at the highly transcribed actin locus. While actin hybrids were readily detected both in HeLa and U2OS cells, RNaseH1 depletion did not affect their abundance, suggesting that RNaseH1 restricts hybrids only at specific loci (Fig. 3b). Total TERRA levels, particularly long TERRA, were slightly diminished in U2OS depleted cells (Fig. 3c), possibly due to compromised transcription when hybrids accumulate, as suggested by our *in vitro* transcription assays (Supplementary Fig. 5). In native FISH experiments performed on interphase nuclei, chromatin-bound TERRA signal increased upon RNaseH1 depletion and it was largely sensitive to RNaseH treatment *in vitro*, confirming that RNaseH1 restricts cellular TERRA hybrids (Fig. 3e).

U2OS depleted cells also accumulated abundant C-rich single-stranded telomeric DNA while the G-rich strand was mildly affected as shown by native telomere restriction fragment (TRF) analysis (Fig. 3f). Likewise, native DNA FISH revealed a faint punctate staining corresponding to C-rich telomeric DNA in siCtrl-transfected U2OS cells and the staining intensity increased upon recombinant RNaseH treatment prior to hybridization, likely due to increased accessibility of the probe. In siRH1c-transfected cells, the C-rich single-stranded DNA (ssDNA) signal was more prominent than in control cells and again it increased significantly upon RNaseH treatment (Fig. 3e,f). As no effect was observed in depleted HeLa cells (Fig. 3c,f), we infer that RNaseH1 regulates hybrids specifically at ALT telomeres thereby maintaining basal levels of C-rich ssDNA, a feature that is not shared with telomerase positive cells. Because telomeric ssDNA exposure might stem from replication defects, we performed IF experiments using antibodies detecting TRF2 and the ssDNA binding protein replication protein A (RPA) phosphorylated at Serine 33 (pSer33), which can be used as a marker for replication stress^28^. We found that pSer33 accumulated at telomeres in U2OS, WI-38 VA13 and GM847 cells depleted for RNaseH1 (Fig. 4 and Supplementary Fig. 7a). We detected sustained induction of pSer33 foci also in HeLa and HT1080 depleted nuclei, yet those foci did not co-localize with telomeres (Fig. 4 and Supplementary Fig. 7a). pSer33 accumulation was not detected in western blots (WBs) of total protein extracts from RNaseH1-depleted U2OS and HeLa cells, while it was readily observed in extracts from cells treated with high doses of the global replication inhibitor hydroxyurea (Fig. 4a). In IF experiments similar HU treatments led to accumulation of pSer33 in a multitude of foci dispersed throughout the entire nucleus rather than in few discrete foci (Fig. 4b). Altogether, these results indicate that RNaseH1 depletion impairs replication at a restricted number of genomic loci that, in U2OS, include telomeres.

### RNaseH1 stabilizes leading-strand telomeres in ALT cells

**Figure 5:**
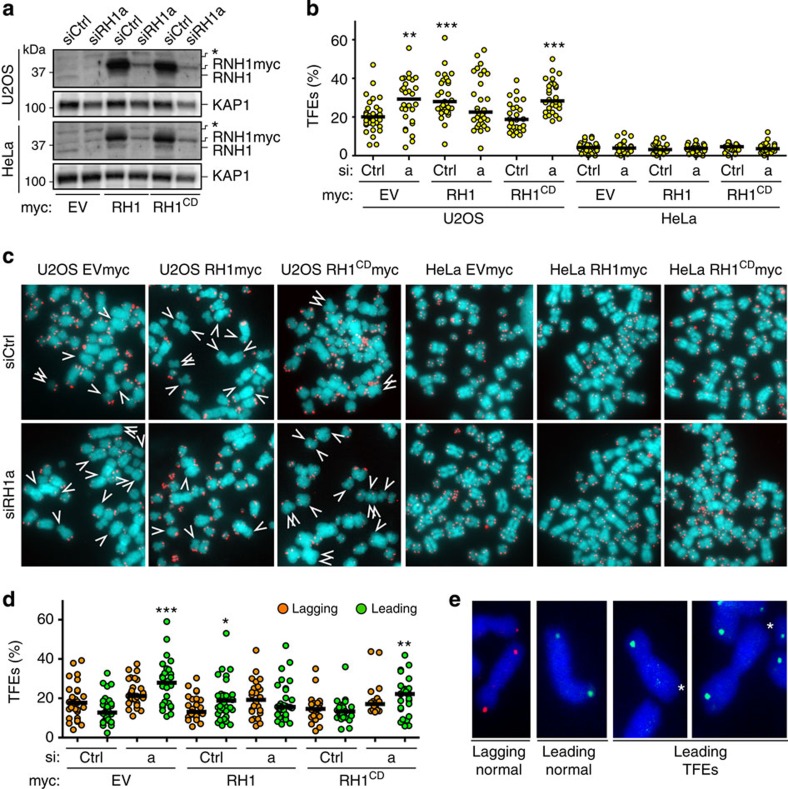
RNaseH1 depletion affects leading-strand telomeres in U2OS cells. (**a**) Western blot analysis of total proteins from U2OS and HeLa cells infected with retroviruses expressing myc-tagged RNaseH1 (RH1) or catalytically dead RNaseH1 (RH1^CD^) or with empty vector control retroviruses (EV) and transfected with the indicated siRNAs. Experiments were performed 6 days after infections and 3 days after siRNA transfections. Endogenous and myc-tagged RNaseH1 proteins were simultaneously detected using anti-RNaseH1 antibodies. The asterisk indicates a cross-reacting band. Total KAP1 was detected to control for loading. (**b**) Quantifications of TFEs in the indicated cells. Each dot represents the fraction of TFEs per chromosome end in one metaphase from two to three independent experiments. Chromosome ends (2,700–3,640) were analyzed for each condition. Black bars indicate medians. *P*-values were computed using the Student’s *t*-test. ***P<0.0001 (indicated sample versus siCtrl-transfected EVmyc sample). (**c**) Examples of FISH experiments performed on metaphase spreads from the indicated cells. Telomeric DNA is in red and DAPI-stained total DNA in blue. Arrows point to TFEs. (**d**) Quantifications of leading and lagging TFEs from CO-FISH experiments performed on the indicated U2OS cells. Each dot represents the fraction of TFEs per chromosome end in one metaphase from two to three independent experiments. Chromosome ends (1,900–3,900) were analyzed for each condition. Black bars indicate medians. *P*-values were computed using the Student’s *t*-test. *P<0.05, **P<0.001, ***P<0.0001 (indicated sample versus siCtrl-transfected EVmyc sample). (**e**) Examples of CO-FISH experiments performed on U2OS cells. Lagging-strand telomeres are in red, leading-strand telomeres are in green and DNA is in blue. Example of normal telomeres and TFEs are shown.

To test the effects of RNAseH1 depletion on telomere stability we developed a siRNA-based complementation system. We stably infected U2OS and HeLa cells with retroviral vectors expressing C-terminally myc-tagged, full-length RNaseH1 (RH1myc) or a catalytically dead variant carrying an Aspartic acid to Alanine substitution at position 145 (ref. 29) (RH1^CD^myc), or with empty vector control retroviruses (EVmyc). Because RNaseH1 expression from retroviruses is driven by the strong cytomegalovirus promoter, infected cells highly overexpressed ectopic RNaseH1 variants (Fig. 5a). To validate the over-expression system we performed S9.6 DIP experiments and found that 10q and 15q telomeric hybrids diminished specifically in RH1myc-expressing U2OS cells. Hybrids did not diminish in U2OS cells expressing RH1^CD^myc or in any HeLa cell sample (Supplementary Fig. 8a). Three days after infections, we transfected cells with siCtrl or siRH1a and 3 days after transfection we performed telomeric DNA FISH experiments. At this time point, siRH1a-transfected cells were largely depleted for endogenous RNaseH1 while the residual levels of RH1myc and RH1^CD^myc were similar or slightly higher as compared with endogenous RNaseH1 in siCtrl-transfected EVmyc cells (Fig. 5a).

Telomeric DNA FISH revealed a striking accumulation of TFEs in RNaseH1-depleted EVmyc U2OS cells. TFE accumulation was reverted in cells expressing RH1myc but not RH1^CD^myc (Fig. 5b,c). Moreover, no change in TFE frequencies was observed in any of the HeLa samples (Fig. 5b,c). Thus, the catalytic activity of RNaseH1 prevents loss of telomeric sequences specifically in ALT cells. We then performed CO-FISH on U2OS cells and found that RNaseH1 depletion specifically induced TFEs at leading-strand chromosome ends, while lagging-strand telomeres were not affected (Fig. 5d,e). As above, TFEs were observed in siRH1a-transfected EVmyc and RH1^CD^myc cells but not RH1myc cells (Fig. 5d,e). We conclude that RNaseH1 depletion engenders unresolved telomeric hybrids specifically at leading-strand telomeres ultimately leading to loss of entire telomeric tracts. Telomere loss is most likely due to intramolecular telomere recombination because C-circles were increased by approximately two and fivefold in U2OS cells transfected with siRH1a and siRH1c, respectively (Fig. 3d). Although the molecular details of how telomeric hybrids promote C-circle excision and TFEs remain to be fully elucidated, it is unlikely that loss of TRF1 or TRF2 have a prominent role, as RNaseH1 depletion did not profoundly alter their density at telomeres (Supplementary Fig. 6d).

### Increased RNaseH1 reduces telomere recombination in ALT cells

**Figure 6:**
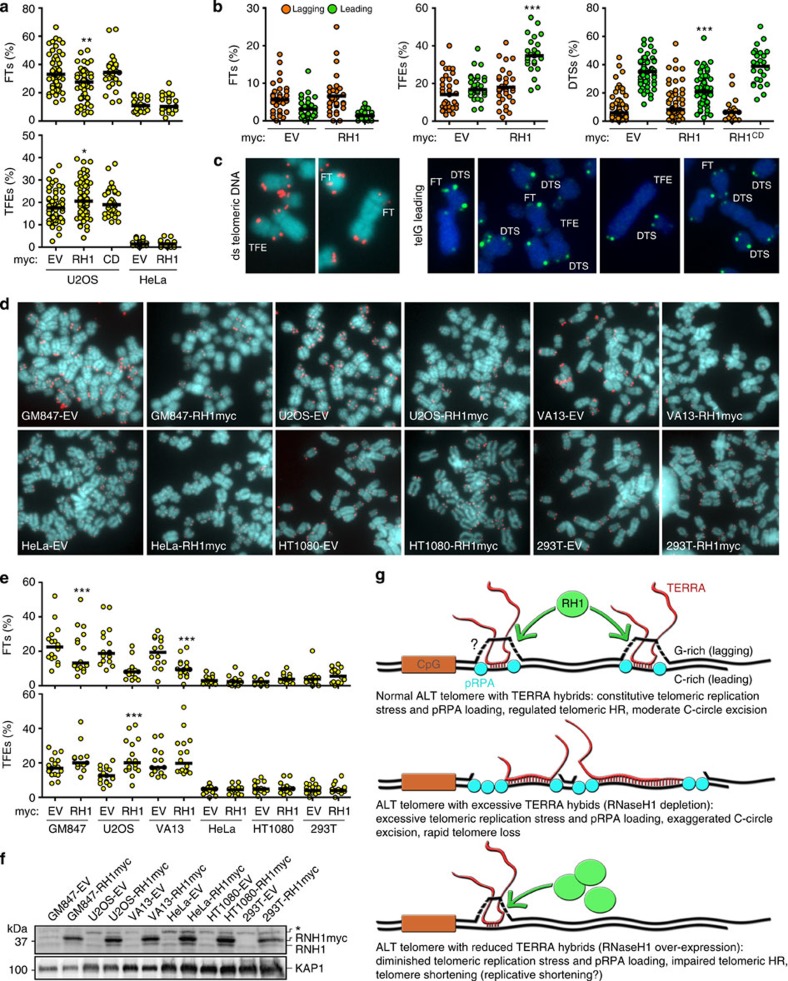
Overexpression of RNaseH1 compromises telomere maintenance in ALT cells. (**a**) Quantifications of fragile telomeres (FTs) and telomere free ends (TFEs) in metaphase spreads from U2OS and HeLa cells infected for 6 days with the indicated retroviruses. Each dot represents the fraction of indicated telomeric aberrations per chromosome end in one metaphase from two to three independent experiments. Chromosome ends (1,200–6,000) were analyzed for each condition. Black bars indicate medians. *P*-values were computed using the Student’s *t*-test. *P<0.05, **P<0.001 (indicated sample versus EVmyc sample). (**b**) Quantifications of leading and lagging TFEs and FTs and of DTSs from CO-FISH experiments performed on U2OS cells infected for 6 days with the indicated retroviruses. Each dot represents the fraction of indicated telomere feature per chromosome end in one metaphase from two to three independent experiments. Chromosome ends (1,900–7,800) were analyzed for each condition. Black bars indicate medians. *P*-values were computed using the Student’s *t*-test. *P<0.05, **P<0.001, ***P<0.0001 (indicated sample versus EVmyc sample). (**c**) Enlarged examples of DNA FISH and CO-FISH experiments on U2OS cells. (**d**) Partial metaphase spreads from the indicated cells infected with retroviruses for 13 days. (**e**) Quantifications of FTs and TFEs on metaphase spreads obtained from cells infected for 13 days with the indicated retroviruses. Chromosome ends (1,200–2,400) were analyzed for each condition. Black bars indicate medians. ***P<0.0001 (RH1myv versus EVmyc). (**f**) Western blot analysis of proteins from cells as in **e** performed with antibodies against RNaseH1 and total KAP1 (loading control). The asterisk indicates a cross-reacting band. (**g**) Consequences of altering RNaseH1 (RH1) cellular levels and telomeric hybrids on ALT telomere maintenance. Question mark indicates displaced G-rich telomeric DNA loops, whose existence remains to be verified.

Our siRNA-based complementation system allowed us also to test whether overexpression of RNaseH1 had any impact on telomere stability. Indeed, U2OS cells overexpressing RH1myc and transfected with siCtrl underwent a global decrease in telomeric signal intensity and accumulated TFEs at leading-strand chromosome ends already at 6 days after infection, while no changes in telomere intensity and TFEs were induced by overexpression of RH1^CD^myc (Fig. 5b–e). We therefore performed more detailed DNA FISH analysis on metaphase spreads from U2OS and HeLa cells overexpressing RNaseH1 variants and not transfected with any siRNA. Overexpression of RH1myc, but not of RH1^CD^myc, induced accumulation of TFEs and a decrease of FTs in U2OS cells while HeLa telomeres remained unaffected upon RH1myc overexpression (Fig. 6a). CO-FISH analysis on U2OS chromosomes showed that RH1myc overexpression induced accumulation of leading-strand TFEs and a mild yet statistically significant decrease in leading-strand FTs (Fig. 6b,c and Supplementary Fig. 7b). Moreover, RH1myc diminished the incidence of leading-strand DTSs at sister telomeres, while not affecting lagging-strand DTSs (Fig. 6b,c). Overexpression of RH1^CD^myc did not affect DTSs (Fig. 6b,c).

We then infected different ALT and telomerase-positive cell lines with EVmyc and RH1myc-expressing retroviruses and analyzed telomere stability 13 days later. Consistent with what was observed in U2OS shortly after infection, prolonged expression of RH1myc diminished global telomere signal intensity as well as the incidence of FTs in U2OS, GM847 and WI-38 VA13, and led to accumulation of TFEs in U2OS. No effect was observed in HeLa, HT1080 and HEK 293T cells (Fig. 6d–f). Finally, overexpression of RH1myc in U2OS cells led to a decrease in the levels of long TERRA molecules upon prolonged culturing and did not alter C-circle formation at any of the tested time-points after infection (Supplementary Fig. 8b,c). Altogether these observations indicated that increasing cellular RNaseH1 levels favours semiconservative replication of telomeric DNA but also reduces the recombinogenicity of leading-strand telomeres, thereby impairing HR-mediated telomere maintenance and causing gradual telomere shortening rather than rampant excision of entire telomeric tracts in the form of C-circles, as it is the case for RNaseH1 depletion.

We also noticed that ~7% of unperturbed U2OS cells had dramatically enlarged nuclei staining positive for pSer33 indicative of sustained replication stress. The large majority of those enlarged nuclei had distinguishable pSer33 foci co-localizing with TRF2 (Supplementary Fig. 7c). Whereas the incidence of these cells was not altered in EVmyc infected cells upon prolonged passaging, RH1myc overexpression essentially eliminated them from the population (Supplementary Fig. 7c). Thus, a relevant fraction of U2OS cells seem to continuously accumulate too many telomeric hybrids and eventually fail to proliferate, most probably due to the activation of intra S-phase checkpoints^30^ signalled by persistent telomere replication stress. These data indicate that telomeric hybrids represent a real threat to cell proliferation and that a minor yet substantial fraction of ALT cells are constantly lost from the population due to aberrant accumulation of such structures.

## Discussion

We have established here that TERRA plays a major role in maintaining ALT telomeres through formation of RNA–DNA hybrid structures with the C-rich telomeric strand. The elevated levels of TERRA typical of ALT cells as well as TERRA localization within APBs, by allowing constant and efficient hybrid formation, might therefore be linked to this specific function of TERRA. We have also revealed that the two telomeric strands do not contribute equally to telomere length maintenance in ALT cells. Rather, the leading-strand telomere appears to be the major substrate templating synthesis of new telomeric material. It is conceivable that the presence of telomeric hybrids on leading-strand telomeres could directly stimulate break-induced replication by exposing C-rich ssDNA patches that would favour invasion of the G-overhang of independent chromosome ends followed by DNA synthesis. The persistent presence of activated RPA at ALT telomeres and its further accumulation when telomeric hybrids are increased might suggest an alternative yet mechanistically overlapping model where unresolved hybrids hinder progression of the replication fork through the leading-strand telomeric template, thereby causing replication fork arrest and generating structures prone to engage in HR.

Our work also reveals that telomeric hybrids, while being necessary to allow telomeric HR and synthesis of new telomeric DNA, can endanger telomere stability if their cellular levels are not properly restricted. Hence, ALT cells need to maintain precise levels of hybrids to support telomeric HR without compromising telomere integrity too severely. In this delicate game, RNaseH1 plays a major role. In cells where the levels of RNaseH1 are too low, excessive amounts of hybrids or a lack of their regulated resolution provoke severe telomere instability, as it is shown by the accumulation of fragile tiTELs upon transcription induction and by the activation of pSer33 and telomere circle excision in RNaseH1-depleted cells (Fig. 6g). On the other side, if RNaseH1 levels are too high, TERRA:telomeric DNA hybrids are insufficient to promote effective telomeric HR and this leads to progressive telomere shortening (Fig. 6g). It will be intriguing to test whether the proliferative potential of ALT cells is affected by long-term RNaseH1 overexpression both in culture and in animal models for cancer development.

Our data beg for different questions that need to be answered to fully understand this aspect of ALT telomere biology. In particular, we now need to clarify the details of how RNaseH1 associates specifically with ALT telomeres. Although we do not exclude that one or more shelterin components could directly promote recruitment of RNAseH1, we rather favour alternative hypotheses that invoke features exclusive of ALT telomeres. One appealing possibility is that RNaseH1 could be recruited as part of a DNA damage response triggered by unresolved hybrids, possibly during specific phases of the cell cycle. The aberrant accumulation of phosphorylated RPA at ALT telomeres when RNAseH1 is depleted might indicate that RPA itself could function in recruiting directly or indirectly RNaseH1. Supporting this hypothesis, it has been shown that ALT cells depleted for RPA accumulate C-rich telomeric ssDNA (ref. 31), similarly to what we observed in cells depleted for RNaseH1. According to this model, RNaseH1 and RPA would therefore be part of an ALT-specific feedback loop regulating telomeric hybrids and HR. Assaying when the cell-cycle RNaseH1 and telomeric hybrids accumulate at ALT telomeres and whether RNaseH1 binding to ALT telomeres is impaired in RPA-compromised cells will help test this hypothesis. Moreover, such a regulatory loop could exist outside telomeres also in non-ALT cells, as it is suggested by the accumulation of non-telomeric pSer33 foci in HeLa cells depleted for RNaseH1.

We expect that our data will strongly impact on our current knowledge of ALT tumours and will pave new ways for a cure. Cancer therapy based on anti-telomerase drugs will not affect ALT tumours and could ultimately select for resistant cells that activate an ALT mechanism. Indeed genetic or chemical inhibition of telomerase led to insurgence of ALT cells in several model systems including cultured human cancer cells, mice and plants^32^,^33^,^34^. On the other side, anti ALT therapeutic approaches based on global suppression of HR—for example by inhibiting HR factors—are dangerous as HR is required for constitutive DNA metabolism. Resolution of TERRA hybrids through enhancement of RNaseH1 activity might become an attractive procedure to fight ALT tumour progression with limited side effects upon treatment of patients.

## Methods

### Cell lines and plasmids

ALT cells, a kind gift from A. Londono Vallejo, were U2OS osteosarcoma cells, WI-38 VA13 *in vitro* SV40-transformed lung fibroblasts and GM847 *in vitro* SV40-transformed skin fibroblasts. Telomerase-positive cells were HeLa (ATCC) and HeLa1.2.11 (kind gift from T. de Lange) cervical carcinoma cells, HT1080 fibrosarcoma cells (ATCC), HEK 293T embryonic kidney cells expressing SV40 large T antigen (ATCC), and HCT116 and DKO human colon carcinoma cells (kind gifts from B. Vogelstein). U2OS-T cells were a kind gift from A. Decottignies. All cell lines were cultured in high glucose D-MEM (Invitrogen) supplemented with 10% TET-free foetal bovine serum (Pan BioTech) and penicillin-streptomycin (Sigma). U2OS tiTEL cells were generated by transfecting linearized tiTEL seeding plasmids^16^ into U2OS cells expressing rTetR (kind gift from P. Silver), followed by selection in 200 μg ml^−1^ hygromycin and clonal selection. tiTEL transcription was induced with 25 ng ml^−1^ dox. Full-length human RNaseH1 complementary DNA (cDNA) was purchased from Origene, C-terminally myc tagged and cloned into the retroviral vector pLHCX (Clontech). Catalytically dead RNaseH1 was obtained by changing the aspartic acid residue at position 145 into alanine (D145A) using the Q5 site directed mutagenesis kit (NEB). Infections were performed according to standard procedures and cells were selected with 200 μg ml^−1^ hygromycin. A stretch of ~800 bp of telomeric DNA (kind gift from E. Gilson) or a 1 kb fragment from Renilla luciferase cDNA were cloned into pCDNA6 (Life Technologies) to generate pTel, pTelR and pLuc plasmids used for *in vitro* transcription with T7 polymerase (NEB) according to the manufacturer’s instructions.

### SiRNAs

Control and target siRNAs were purchased from Integrated DNA Technologies. Target sequences were: 5′-ACAAACCAAAGAGCGGAAATTCATG-3′ (siRH1a), 5′-TCCTTTAAATGTAGGCATTAGACTT-3′ (siRH1c), 5′-GCAACAAGACCTTAATAAGAAAGAA-3′ (siTRF1), 5′-AGAATCCCAAAGTACCCAAAGGCAA-3′ (siTRF2). Cells were transfected with 20–25 nM siRNAs using Lipofectamine RNAiMAX (Invitrogen).

### Nucleic acids analysis

Total RNA was isolated using the TRIzol reagent (Invitrogen) and genomic DNA was prepared using phenol/chloroform extraction. For northern blotting 10–15 μg RNA were electrophoresed in 1.2% agarose formaldehyde gels. To detect long TERRA species, gels were treated with 50 mM NaOH, 1.5 M NaCl for 10 min and transferred onto nylon membranes^6^,^8^,^13^. A strand-specific radiolabelled telomeric probe was used to detect TERRA (refs 6, 8, 13). Hybridizations were performed at 55 °C for 18 h. TRF analysis was performed using genomic DNA digested with *Hinf*I and *Rsa*I. TERRA promoter methylation analysis was performed using genomic DNA digested with either *Msp*I or *Hpa*II. Restriction fragments were electrophoresed in 0.6% (for TRF analysis) or 1.2% (for methylation analysis) agarose gels. For TRF analysis, gels were dried and hybridized in native conditions at 42 °C using radiolabelled oligonucleotides detecting C-rich or G-rich telomeric sequences. After signal detection, gels were denatured and re-hybridized as for northern blotting. For promoter methylation analysis, DNA was transferred to nylon membranes and hybridized to TERRA promoter specific probes^8^ at 60 °C for 18 h. C-circle assays^14^ were performed using 500 ng of *Hinf*I and *Rsa*I digested genomic DNA and Ф29 DNA polymerase (NEB). Amplification products were dot-blotted onto nylon membranes and hybridized as for northern blot analysis in native conditions. Radioactive signals were detected using a Typhoon FLA 9000 imager (GE Healthcare). For real-time reverse transcription PCR, 5 μg of total RNA were reverse transcribed using Superscript III reverse transcriptase (Invitrogen) with a 5′-(CCCTAA)_5_-3′ oligonucleotide. cDNA was PCR amplified using a Rotor-Gene Q instrument (Qiagen), the LightCycler 480 SYBR Green I Master mix (Roche) and the following primer pairs: 5′-GAATCCTGCGCACCGAGAT-3′ and 5′-CTGCACTTGAACCCTGCAATAC-3′ for 10qTERRA; 5′-AGAATCTCACGCAGGCAGTT-3′ and 5′-CCAGGGATTTCAGTCGATGT-3′ for tiTERRA.

### IF, WBs and ChIP assays

Indirect IF, WB and ChIP assays were carried out according to standard protocols. Antibodies used were a rabbit polyclonal anti-TRF2 (Novus Biologicals, NB110-57130, IF dilution 1:500, WB 1:2,000), a rabbit polyclonal anti-TRF1 (kind gift from J. Karlseder, WB 1:1,000), a rabbit polyclonal crude serum anti-TRF1 (kind gift from J. Lingner, ChIP 1:500), the mouse monoclonal S9.6 (kind gift from S. Leppla, IF 1:1,000), rabbit polyclonals anti-RNAseH1 and anti-Lamin B1 (GeneTex GTX117624, WB 1:500; GTX103292S, WB 1:1,000), rabbit polyclonals against total RPA32, pSer33, total KAP1, phosphorylated serine S2 or serine S5 from human RNAPII C-terminal domain (Bethyl Laboratories A300-244A, WB 1:5,000; A300-246A WB and IF 1:1,000; A300-274A, WB 1:5,000; A300–654A, ChIP 1:500; A300–655A, ChIP 1:500), mouse monoclonals anti-PCNA and PML (Santa Cruz Biotechnology sc-56, WB 1:5,000; sc-966, IF 1:250).

### DNA immunoprecipitation

Cells were cultured until ~70% confluent, harvested on ice and lysed in 1 ml of RA1 buffer (Macherey-Nagel) containing 10 μl of β-mercaptoethanol. Samples were mixed with 700 μl of phenol:chloroform:isoamyl alcohol (25:24:1 saturated with 10 mM Tris-Cl pH 8.0, 1 mM EDTA), mixed by vortexing and centrifuged (13,000*g*, 15 min, 4 °C). Upper phases were transferred to new tubes and nucleic acids were precipitated with 1 ml of isopropanol followed by centrifugation (13,000*g*, 20 min, 4 °C). Pellets were washed with 70% ethanol, air-dried, re-suspended in 200 μl of Tris-EDTA and sonicated with a Bioruptor (Diagenode) to obtain 100–500 bp long fragments. Ten micrograms of sheared nucleic acids were diluted in 1 ml of IP buffer (0.1% SDS, 1% Triton X‐100, 10 mM HEPES pH 7.7, 0.1% sodium deoxycholate, 275 mM NaCl) and incubated for 2 h on a rotating wheel at 4 °C in presence of 1 μg of S9.6 antibody and 20 μl of protein G sepharose beads (GE Healthcare) blocked with *Escherichia coli* DNA and bovine serum albumin. Beads were washed four times with IP buffer and bound nucleic acids were isolated using the Wizard SV gel and PCR clean-up kit (Promega). Real-time PCRs for 10q, 15q and XpYp subtelomeres and tiTELs were performed as previously described^6^,^8^. In control experiments, nucleic acids were treated with 100 U of RNaseH (NEB) for 10 h prior to IP.

### DNA and RNA FISH

TERRA was detected with a mixture of 1–5 kb long telomeric DNA fragments strand-specifically labelled by random priming with Cy3-dCTP, dTTP and dATP (Perkin Elmer) according to previously published protocols^6^. The following probes were used for DNA FISH and CO-FISH experiments: lacO PNA probe, 5′-FITC-OO-AATTGTTATCCGCTCACAATTC-3′ (Bio-Synthesis Inc.); TelC PNA probe 5′-Cy3-00-CCCTAACCCTAACCCTAA-3′ (Panagene); TelG LNA probe 5′-Tex615-GGGT*TAGGG*T*TAG*GGTTAGGG*T*TAGGG*T*TAGGG*TTA-3′ (Exiqon, asterisks indicate LNA nucleotides). Cells were treated with 200 ng ml^−1^ Colcemid for 2–3 h, harvested and incubated in 0.075 M KCl at 37 °C for 9 min. Chromosomes were fixed in cold methanol/acetic acid (3:1) and spread on glass slides. DNA FISH was performed as previously described^6^,^16^. CO-FISH was performed according to published protocols^14^ with the following modifications. Cells were incubated with 7.5 mM BrdU for 16 h prior to Colcemid treatment. TelG LNA probe hybridization was performed in 30% formamide, 2 × SSC for 3 h at room temperature (RT) and post-hybridization washes were in 2 × SSC at RT. For native FISH experiments on interphase nuclei, cells grown on coverslips were washed with ice-cold 1 × PBS and soluble cellular material was extracted with CSK buffer (100 mM NaCl, 300 mM sucrose, 3 mM MgCl_2_, 0.5% Triton X, 10 mM PIPES pH 6.8) for 7 min on ice. Cells were then fixed in 3.7% formaldehyde for 10 min, washed in 1 × PBS and permeabilized with CSK buffer for 5 min at RT. RNaseH (NEB) treatments were done by incubating slides with 50 U of RNaseH or with buffer only for 6 h 37 °C in a humidified chamber. Hybridizations were performed for 3 h at RT for the TelG LNA probe or overnight at 37 °C with the TeloA probe. Images were acquired with an Olympus IX 81 microscope and statistical analyses were carried out using Excel (Microsoft) or Prism (Graph Pad).

### IF/RNA FISH

Cells grown on coverslips were treated as described above for native FISH with the following additional steps. After the second permeabilization in CSK buffer, cells were incubated in ice-cold methanol at −20 °C for 20 min followed by washes in 1 × PBS. Cells were blocked in 5 mg ml^−1^ BSA in 1 × PBS, 0.05% Tween-20 and 2 mM Glycine for 30 min at RT. After primary and secondary antibody staining, cells were fixed with 3.7% formaldehyde for 10 min at RT and blocked again in 5 mg ml^−1^ BSA in 1 × PBS, 0.05% Tween-20 and 2 mM Glycine for 15 min at RT. Samples were incubated in 2 × SSC for 5 min at RT and then hybridized for 2 h at 37 °C in a humid chamber with 200 ng of Cy5-conjugated TelC oligonucleotide probe in hybridization mix consisting of 1 part of 20 × SSC, 2 parts of 10 mg ml^−1^ BSA, 2 parts of 50% dextran sulphate and 5 parts of deionized formamide. Post-hybridization washes and image acquisition were as described for TERRA FISH. Image analysis to quantitate TERRA intensity were performed using ImageJ.

### Three-dimensional structured illumination microscopy

Images were acquired on a DeltaVision OMX V4 Blaze (Applied Precision Imaging/GE Healthcare) equipped with a × 60/1.42NA PlanApo oil immersion objective (Olympus). Super-resolution stacks were reconstructed using a Wiener filter constant of 0.001 and channel specific measured optical transfer functions. Image registration was performed in softWoRx 6.0 with alignment parameters obtained from a TetraSpeck bead 0.2 μm in diameter (Invitrogen, Molecular probes). The voxel size of the reconstructed image is 40 nm in *xy* and 125 nm in *z*. The reconstructed and registered images were three-dimensional rendered (with interpolation) in Imaris6 (Bitplane) and information surfaces were created at a detailed level of 0.01 μm.

## Author contributions

R.A., Y.L. and C.M.A. conceived the rationale of the experiments. R.A, Y.L., H.W., C.M.B. and C.M.A. performed the experiments and analyzed the data. T.S. performed the super-resolution experiments and analyzed the data. R.A. and C.M.A wrote the paper.

## Additional information

**How to cite this article:** Arora, R. *et al.* RNaseH1 regulates TERRA-telomeric DNA hybrids and telomere maintenance in ALT tumour cells. *Nat. Commun.* 5:5220 doi: 10.1038/ncomms6220 (2014).

## Supplementary Material

Supplementary Figures Supplementary Figures 1-8
