# Supplementary material for: RNaseH1 regulates TERRA-telomeric DNA hybrids and telomere maintenance in ALT tumour cells
Source: Nat Commun. 2014 Oct 21;5:5220. doi: 10.1038/ncomms6220 (PMC4218956; doi:10.1038/ncomms6220)
Supplement: Supplementary Figures — 1-8 [file ncomms6220-s1.pdf]

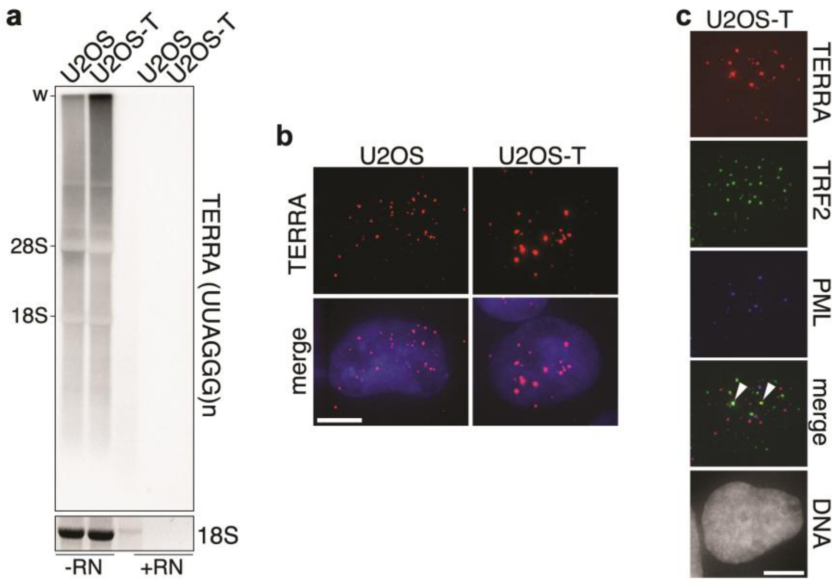

d

	Nuclei	TERRA foci [median]	APBs [median]	TERRA in APBs [%]	APBs with TERRA [%]
**U2OS**	40	46	6	8	60
**U2OS-T**	40	32	8	4	12
**VA13**	26	20	20	37	42

Supplementary Figure 1. TERRA expression and localization in ALT cells. (**a**) TERRA northern blot hybridizations of RNA from U2OS parental cells and U2OS cells ectopically expressing telomerase (U2OS-T). RNA was pre-treated with RNaseA (+RN) or left untreated. Ethidium bromide stained 18S rRNA is shown to control for loading. (**b**) Examples of TERRA FISH in U2OS and U2OS-T cells. TERRA is shown in red, DAPI stained DNA in blue. Scale bar: 9 μm. (**c**) IF/FISH experiments in U2OS and U2OS-T cells. TERRA is in red, TRF2 in green and PML in blue. In the merge panel, arrowheads point to nuclear foci where the three factors co-localize. Scale bar: 9 μm. (**d**) Quantification of TERRA co-localization with APBs in the indicated cell lines. APBs were defined as nuclear foci containing both TRF2 and PML. Cumulative numbers of nuclei scored from two independent experiments are indicated in the first column.

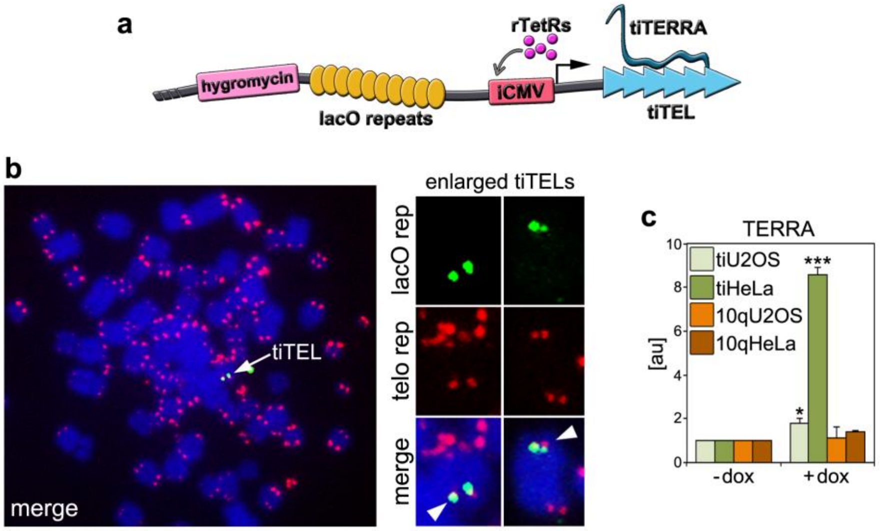

Supplementary Figure 2. Characterization of the U2OS tiTEL cell line. (**a**) Schematic representation of a tiTEL. An inducible cytomegalovirus (iCMV) promoter is inserted immediately upstream of the 'transcriptionally inducible telomere' (tiTEL), which is transcribed into 'transcriptionally inducible TERRA' (tiTERRA). The promoter is preceded by an array of lacO repeats, used to detect tiTEL-containing chromosome ends in FISH experiments, and by a hygromycin resistance cassette, used to select positively transfected clones. The parental U2OS cell line stably expresses the reverse TET repressor (rTetRs). (**b**) Metaphase spread from tiTEL U2OS cells stained for telomeric DNA (in red) and lacO DNA (green). DNA is counterstained with DAPI (blue). The arrow indicates the unique chromosome comprising the tiTEL. Examples of enlarged tiTELs are shown on the right and indicated by arrowheads. (**c**) HeLa and U2OS tiTEL cells were treated with doxycycline for 48 hours (+dox) or left untreated (-dox) and tiTERRA expression was measured by real time RT-PCR using a pair of oligonucleotides comprised between the iCMV promoter and the telomeric tract. Endogenous TERRA from 10q chromosome ends remained unaffected, excluding secondary effects associated to dox treatments. Bars and error bars are averages and s.d. from at least three independent experiments; -dox values were set to 1. P-values were computed using the Student's t-Test. *P< 0.05, ***P< 0.0001 (+dox *vs* -dox).

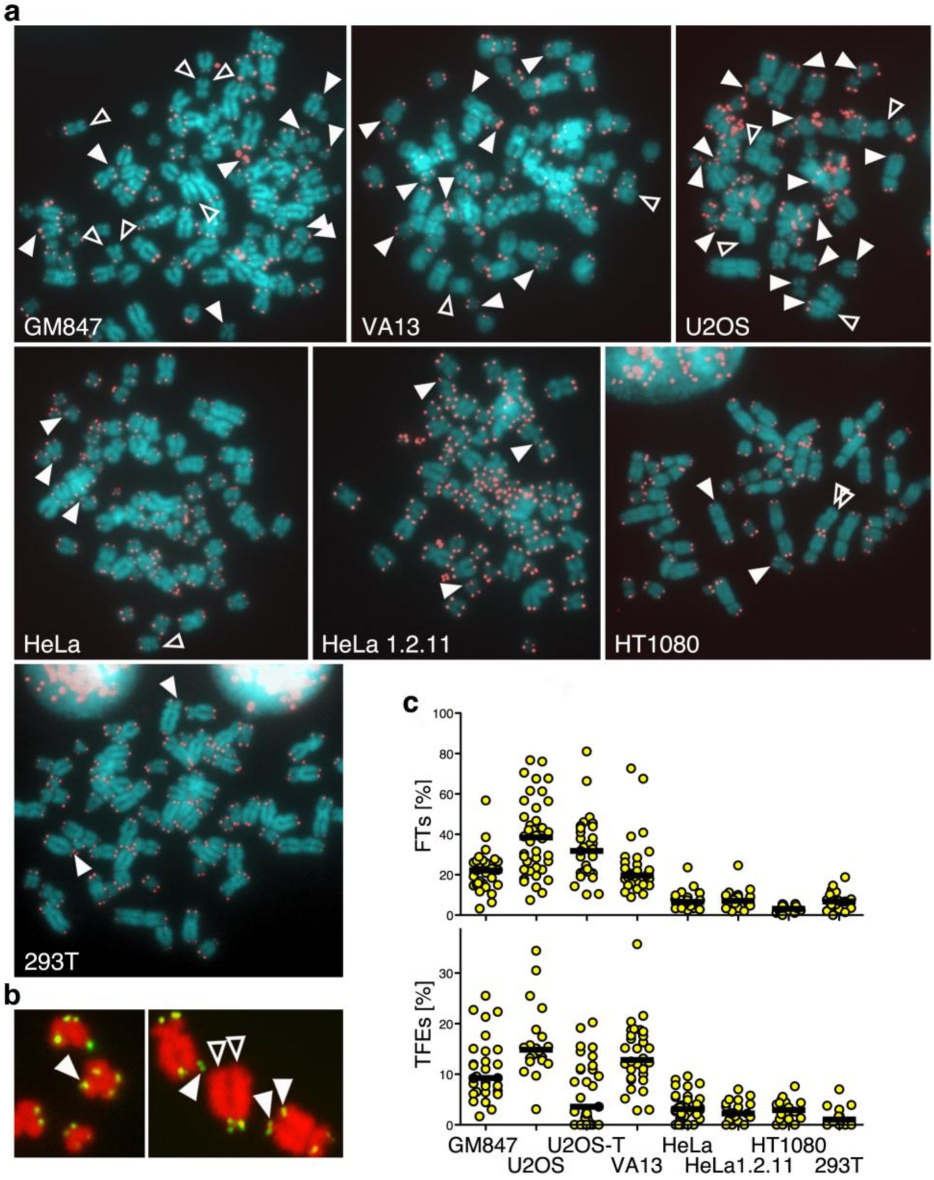

Supplementary Figure 3. ALT cells have unstable telomeres. (**a**) Metaphase spreads from the indicated cell lines were stained for telomeric DNA (red) and counterstained with DAPI (blue). (**b**) Enlarged examples of chromosomes with fragile telomeres (FTs, white arrowheads) and telomere free ends (TFEs, empty arrowheads) taken from U2OS spreads. (**c**) Quantifications of telomeric aberrations in the indicated cell lines. U2OS-T is a U2OS-derived cell line ectopically expressing telomerase. Each dot represents the fraction of FTs or TFEs per chromosome end in one metaphase from 2 to 3 independent experiments. 1600 to 5100 chromosome ends were analyzed for each condition. Black bars indicate medians.

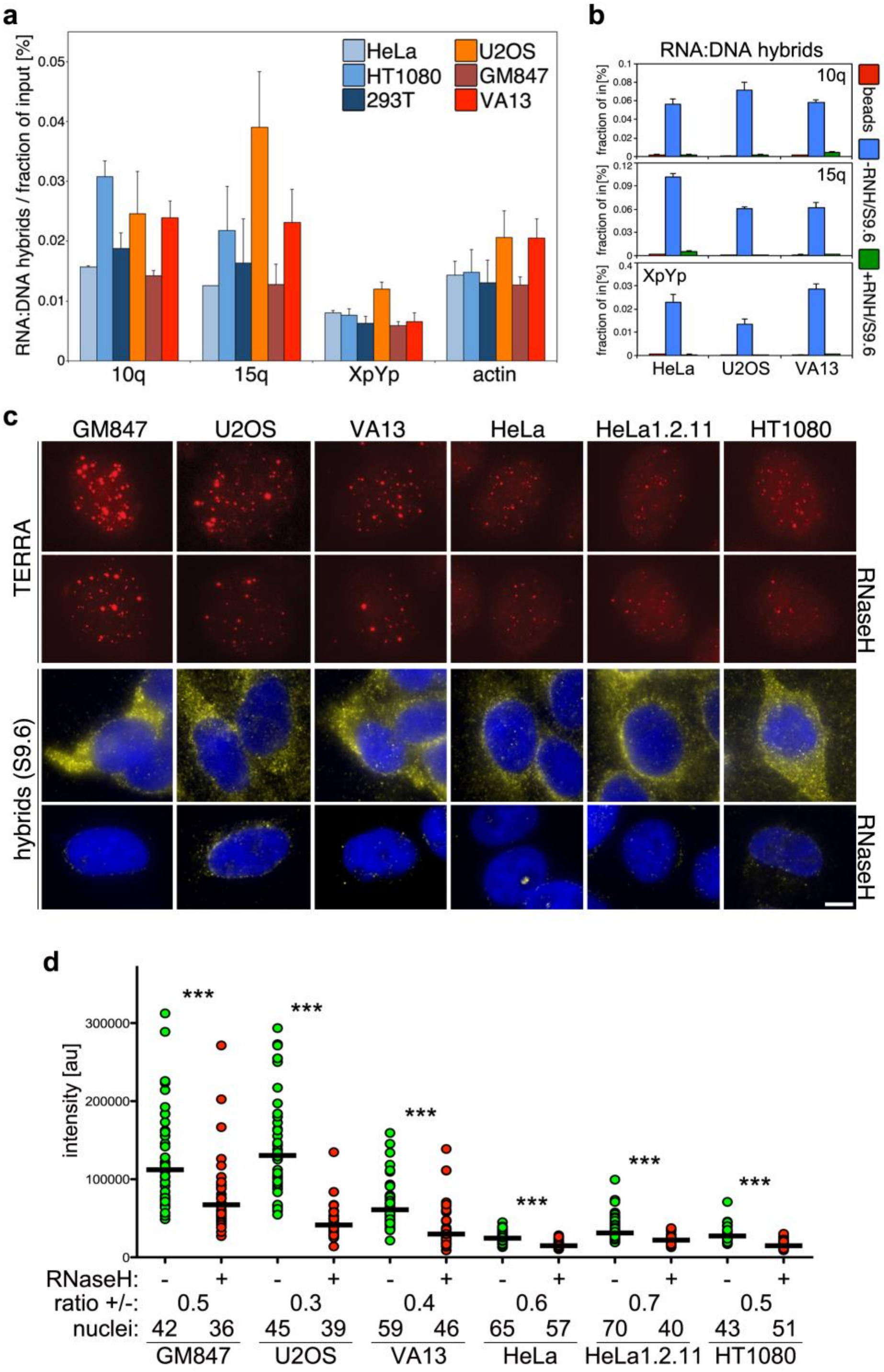

Supplementary Figure 4. RNA-DNA hybrids are present at human chromosome ends. (**a**) DIP experiments were performed using the S9.6 antibody and nucleic acids from the indicated cell lines, followed by quantitative real time PCR using primer pairs amplifying regions close to the telomeric tracts of the indicated chromosome ends or the actin locus. Hybrids are expressed as fractions of the input material after subtraction of the values corresponding to control immunoprecipitation performed with only beads. Bars

and error bars are averages and s.d. from at least three experiments. (**b**) The same experiments shown in **a** were performed using nucleic acids previously treated for 10 hours with RNaseH *in vitro* (+RNH/S9.6) or with reaction buffer (-RNH/S9.6) to control for antibody specificity. Bars and error bars are averages and s.d. from at least three experiments. (**c**) TERRA FISH was performed on fixed cells treated for 6 hours with RNaseH *in vitro* or with reaction buffer. TERRA signal is shown in red. To control for RNaseH digestion, cells were also stained using the S9.6 antibody (yellow) and counterstained with DAPI (blue). Scale bar: 9 μm. (**d**) Quantifications of experiments shown in **c**. Each dot represents the total TERRA signal intensity recorded for one nucleus. Black bars indicate medians. The ratio between the median intensity of RNaseH treated samples and samples left untreated (ratio +/-) and the total number of analyzed nuclei are indicated. P-values were computed using the Student's t-Test. ***$P < 0.0001$ (RNaseH treated *vs* untreated).

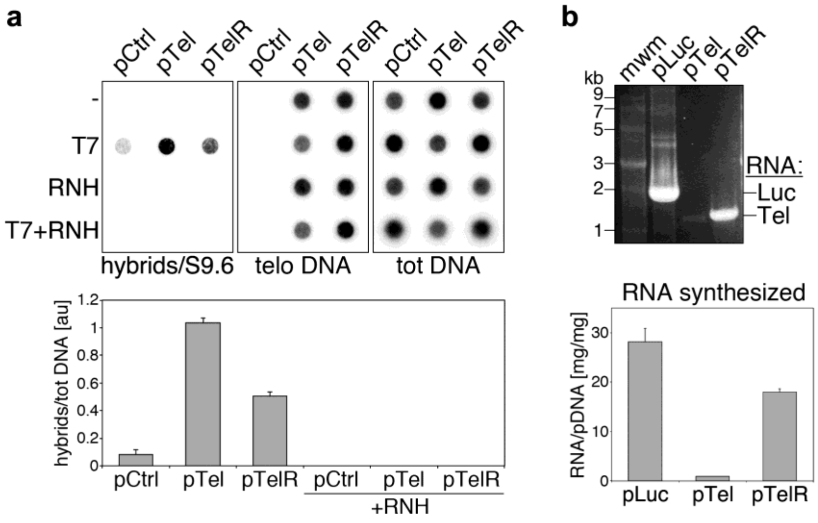

Supplementary Figure 5. *In vitro* transcription of telomeric DNA produces abundant RNA-DNA hybrids. (**a**) T7 polymerase transcription was performed *in vitro* using as templates circular empty vector plasmids (pCtrl) or plasmids containing an ~800 bp long telomeric tract leading to transcription of TERRA-like molecules (pTel) or antisense TERRA-like molecules (pTelR). Single stranded RNA was digested and reaction products were incubated with RNaseH (T7+RNH) or buffer only for 2 hours before dot blotting onto nylon membranes. RNA-DNA hybrids were immunodetected using the S9.6 antibody. Membranes were then denatured and hybridized successively to telomeric probes to detect telomeric DNA and to plasmid probes to detect total plasmid DNA. Control reactions without T7 (-) were also included. The graph at the bottom indicates quantifications of S9.6 signals after normalization using the corresponding signal detected with total DNA probes. Bars and error bars are averages and s.d. from 5 independent experiments; pTEL values were set to 1. (**b**) Linearized pTel and pTelR plasmids were T7 transcribed and total RNA was purified. A linearized plasmid containing a Luciferase cDNA (pLuc) was used as a control template. Equal reaction volumes were loaded on an agarose gel and stained with ethidium bromide. RNA was quantified and yields are plotted in the graph at the bottom. Bars and error bars are averages and s.d. from 5 independent experiments; pTel values were set to 1.

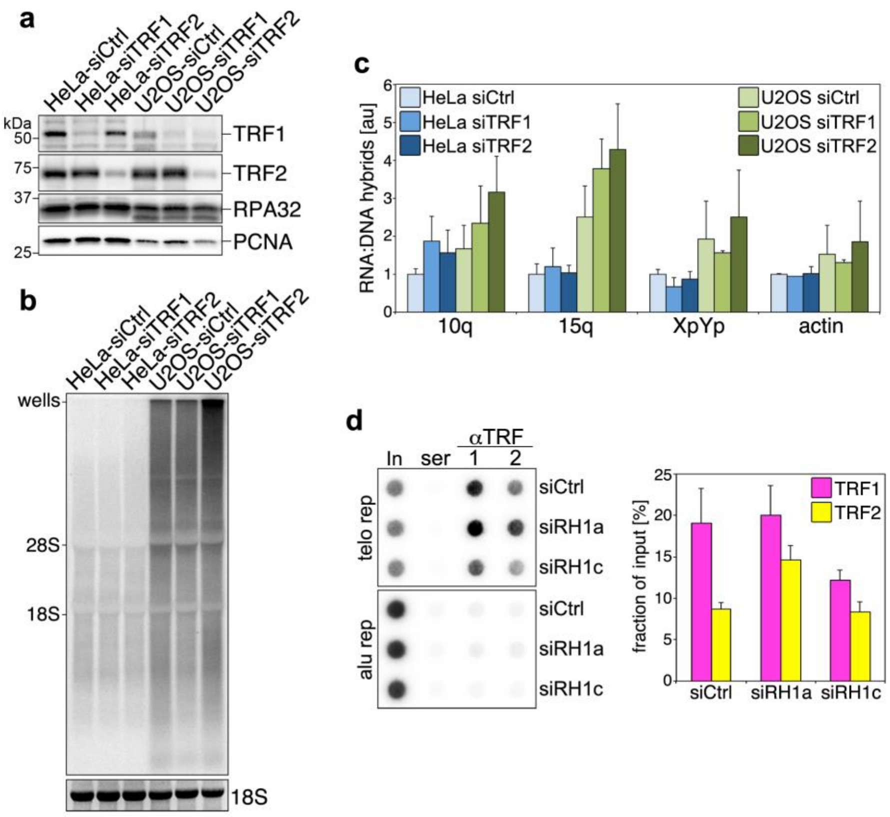

Supplementary Figure 6. Depletion of TRF1 and TRF2 does not affect telomeric hybrid levels substantially. (**a**) HeLa and U2OS cells were transfected with siRNAs against TRF1 and TRF2 or with control siRNAs (siCtrl). 72 hours later protein extracts were collected and TRF1 and TRF2 depletions were validated by western blot analysis. Total RPA32 and PCNA were used as loading controls. (**b**) TERRA northern blot using total RNA from TRF1 and TRF2 depleted cells. (**c**) Quantification of telomeric hybrids from the indicated chromosome ends and actin locus measured in S9.6 DIP experiments using cells transfected with the indicated siRNAs. Hybrids are expressed as fractions of the input material after subtraction of the values corresponding to control immunoprecipitation with only beads. SiCtrl was set to 1. Bars and error bars are averages and s.d. from at least three experiments. (**d**) U2OS cells were transfected with siCtrl and siRH1a and siRH1c and 72 hours later cross-linked chromatin extracts were prepared and subjected to ChIP using crude sera against TRF1 and TRF2 or an unrelated control serum (ser). 10% of input (In) and immunoprecipitated DNA were dot-blotted and hybridized consecutively with telomeric and alu repeat probes. Quantifications of immunoprecipitated telomeric DNA expressed as fraction of input telomeric DNA are on the right. Bars and error bars are averages and s.d. from three experiments.

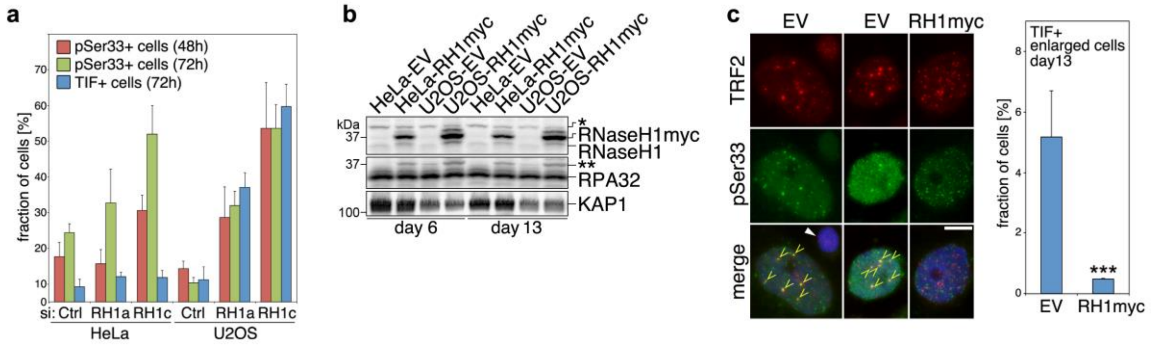

Supplementary Figure 7. RPA response upon RNaseH1 depletion and over-expression. (**a**) Quantifications of immunofluorescence experiments performed on HeLa and U2OS cells transfected with the indicated siRNAs. pSer33+ cells are cells with at least 5 distinct pSer33 foci, TIF+ cells are cells with at least 5 pSer33 foci co-localizing with TRF2. Bars and error bars are averages and s.d. from three experiments. At least 100 cells were counted in each experiment. (**b**) U2OS and HeLa cells were infected with retroviruses expressing RH1myc or empty vector (EV) control retroviruses and protein extracts were collected 6 and 13 days after infection. Western blot analysis was performed using antibodies against RNaseH1 and total KAP1 (loading control). The asterisk indicates a cross-reacting band. (**c**) Examples of U2OS cells with enlarged nuclei and RPA phosphorylated at Serine 33 foci (pSer33, green) at telomeres (visualized with anti TRF2 antibodies, red). Yellow arrows points to co-localizing pSer33 and TRF2 foci (TIFs). The white arrowhead points to an average sized nucleus. Scale bar: 27 μm. Quantifications of large cells with at least 5 TIFs (TIF+) are shown on the right for U2OS cells 13 days after infection. Bars and error bars are averages and s.d. from three experiments. At least 100 nuclei were scored in each experiment. P-values were computed using the Student's t-Test. ***$P < 0.0001$.

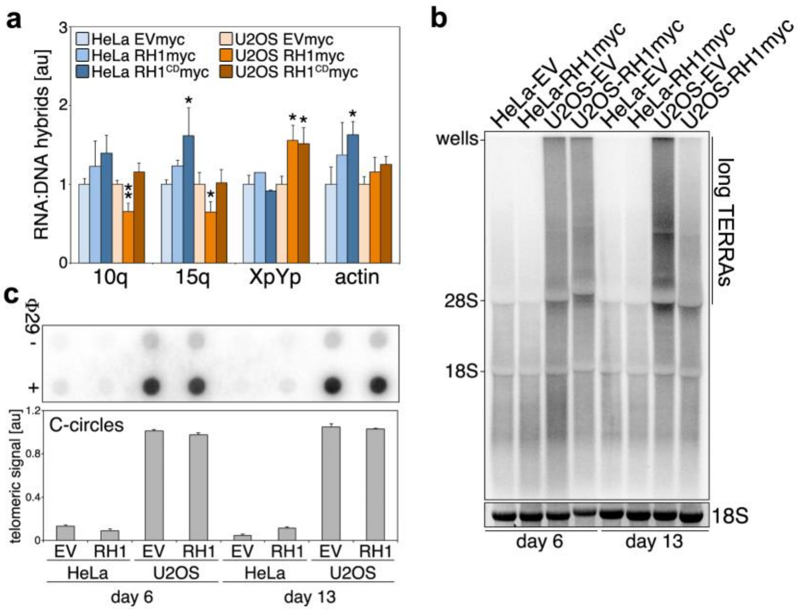

Supplementary Figure 8. Effects of RNaseH1 over-expression on TERRA, telomeric hybrids and C-circles. (**a**) Quantification of telomeric hybrids from the indicated chromosome ends and actin locus measured in S9.6 DIP experiments using HeLa and U2OS cells infected for 6 days with retroviruses expressing RH1myc or empty vector (EV) control. Hybrids are expressed as fractions of the input material after subtraction of the values corresponding to control immunoprecipitation with only beads. EV values were set to 1. Bars and error bars are averages and s.d. from two experiments. P-values were computed using the Student's t-Test. **P*< 0.05. (**b**) TERRA northern blot using total RNA from HeLa and U2OS infected cells. (**c**) C-circle assays in HeLa and U2OS infected cells. Dot blots were hybridized with a C-rich telomeric probe in native conditions. Negative controls are reactions performed in absence of Φ29 enzyme (upper lane). Quantifications of C-circle signals are at the bottom where values for EV infected U2OS were set to 1. Bars and error bars are averages and s.d. from three experiments.
